# ﻿*Branchiostegussanae*, a new species of deepwater tilefish (Eupercaria, Branchiostegidae) from the South China Sea

**DOI:** 10.3897/zookeys.1227.130512

**Published:** 2025-02-11

**Authors:** Haochen Huang, Jingxuan Chen, Zhixin Ke, Chi Zhang

**Affiliations:** 1 Key Laboratory of Tropical Marine Bio-resources and Ecology, South China Sea Institute of Oceanology, Chinese Academy of Sciences, Guangzhou, 510301, China South China Sea Institute of Oceanology, Chinese Academy of Sciences Guangzhou China; 2 University of Chinese Academy of Sciences, Beijing, 100049, China University of Chinese Academy of Sciences Beijing China; 3 College of Life Sciences, Zhejiang University, Hangzhou, 310058, China Zhejiang University Hangzhou China; 4 Guangdong Provincial Key Laboratory of Applied Marine Biology, Guangzhou, 510301, China Guangdong Provincial Key Laboratory of Applied Marine Biology Guangzhou China; 5 Fisheries College, Ocean University of China, Qingdao 266003, China Ocean University of China Qingdao China

**Keywords:** Bottom fish, Hainan, Malacanthidae, phylogeny, taxonomy

## Abstract

A new species of deepwater tilefish, *Branchiostegussanae***sp. nov.**, is described based on five specimens collected from the area between the Xisha Islands and Hainan Island, China. This species can be distinguished from congeners by its unique cheek marker and a combination of characteristics. Among the tilefish species known to be distributed in the South China Sea, this species is the only one with vertical stripes on the body. Based on the *COI*, *CytB*, and *12S* sequences, a maximum-likelihood phylogenetic tree shows that the *B.sanae***sp. nov.** forms a separate clade and is the sister group to the clade consisting of *B.sawakinensis*, *B.albus*, *B.argentatus*, *B.biendong*, *B.japonicus*, *B.auratus*, and *B.okinawaensis*. A key to the genus *Branchiostegus* is also compiled.

## ﻿Introduction

Deepwater tilefishes (family Branchiostegidae) are comprised of three genera: *Branchiostegus* Rafinesque, 1815; *Caulolatilus* Gill, 1862; and *Lopholatilus* Goode & Bean, 1879 (Dooley 1978; WoRMS 2024). This family includes 30 species, but only one species, *Branchiostegusbiendong* Hiramatsu, Vinh & Endo, 2019, was described in the last decade (Nelson et al. 2016; Hiramatsu et al. 2019; Van der Laan and Fricke 2024). Most deepwater tilefish species belong to the genus *Branchiostegus*, which can be found from warm temperate to tropical oceans. Generally, the genus *Branchiostegus* can be identified by the following characteristics: nearly quadriform head, no barbel, preoperculum without a strong spine, moderately elongate and compressed body, presence of predorsal ridge with unraised posterior end, counts of fin-rays (dorsal fin VI–VII, 15–16, pelvic fin I, 5, anal fin I–II, 11–13), and 40–72 intermittent pored lateral-line scales (Dooley 1978; Dooley and Kailola 1988). These fishes inhabit sandy and muddy bottoms on the edges of continental and oceanic plates, usually at depths of 20–200 m (Dooley 1978; Hiramatsu et al. 2019). Up to date, 16 valid species and two nomina dubia are known for this genus, which are primarily distributed in the Indo-West Pacific (Table 1). Only one species (*B.semifasciatus*) was found in the eastern Atlantic.

**Table 1.** A list of 16 valid species and two nomina dubia in the genus *Branchiostegus*.

Species	Source
*B.albus* Dooley, 1978	Dooley 1978; Dooley and Kailola 1988
*B.argentatus* (Cuvier, 1830)	Dooley and Kailola 1988
*B.auratus* (Kishinouye, 1907)	Kishinouye 1907; Dooley and Kailola 1988
*B.australiensis* Dooley & Kailola, 1988	Dooley and Kailola 1988; Bray 2021
*B.biendong* Hiramatsu, Vinh & Endo, 2019	Hiramatsu et al. 2019
*B.doliatus* (Cuvier, 1830)	Dooley 1978; Dooley and Kailola 1988
*B.gloerfelti* Dooley & Kailola, 1988	Dooley and Kailola 1988
*B.hedlandensis* Dooley & Kailola, 1988	Dooley and Kailola 1988
*B.ilocanus* Herre, 1928*	Dooley 1978; Dooley and Kailola 1988
*B.japonicus* (Houttuyn, 1782)	Dooley 1978; Dooley and Kailola 1988
*B.okinawaensis* Hiramatsu & Yoshino, 2012	Hiramatsu and Yoshino 2012
*B.paxtoni* Dooley &Kailola, 1988	Dooley and Kailola 1988
*B.saitoi* Dooley &Iwatsuki, 2012	Dooley and Iwatsuki 2012
*B.sawakinensis* Amirthalingam, 1969	Dooley 1978; Dooley and Rau 1982; Muhammad and El-Mahdy 2022
*B.semifasciatus* (Norman, 1931)	Dooley 1978; Dooley and Kailola 1988
*B.serratus* Dooley &Paxton, 1975	Dooley and Paxton 1975; Dooley 1978; Dooley and Kailola 1988
*B.vittatus* Herre, 1926*	Dooley 1978; Dooley and Kailola 1988
*B.wardi* Whitley, 1932	Dooley 1978; Dooley and Kailola 1988

*Nomen dubium.

In 2021, we noticed that some deepwater tilefish individuals had a unique cheek pattern in some online seafood markets. This unique pattern sets them apart from other deepwater tilefishes. Afterwards, we collected some specimens of this deepwater tilefish and cross-validated the fishing area from various fishermen in March 2023. Hence, we describe this new species based on these specimens. A key to species of the genus *Branchiostegus* is also compiled, and the genus *Branchiostegus* in China is briefly reviewed.

## ﻿Materials and methods

### ﻿Specimen collection and morphological data

All specimens in this study were purchased from fishermen and originated from legitimate commercial fishing activities. Morphological counts and measurements followed Hiramatsu et al. (2019). One holotype and four paratypes are designated. The holotype was deposited at the Marine Biological Museum, Institute of Oceanology, Chinese Academy of Sciences (**IOCAS**), Qingdao, China. Four paratypes were deposited in four research departments: Institute of Zoology, Chinese Academy of Sciences (**IOZ**), Beijing, China; Marine Biodiversity Collection of South China Sea, South China Sea Institute of Oceanology, Chinese Academy of Sciences (**SCSIO**), Guangzhou, China; Shanghai Natural History Museum (**SNHM**), Shanghai, China; and Zoological Specimens Room, Zhejiang University (**ZJU**), Hangzhou, China. Non-type specimens are deposited at the ZJU and Fisheries Resource Biology Laboratory, Fisheries College, Ocean University of China, Qingdao, China All specimens are preserved in 75% alcohol.

### ﻿Molecular data, phylogenetic analysis, and DNA-based species delimitation

The authors used gills for all DNA extractions. Genomic DNA was extracted using the Tissue DNA Kit (DP324-02, TIANGEN Biotech (Beijing) Co., Ltd). The genomic DNA was dissolved in 100 ml of ultra-pure water and stored at 4 °C. The mitochondrial fragments of the regions *COI*, *CytB*, and *12S* were amplified. *COI* was amplified using F1/F2 and R1/R2 (Ward et al. 2005), *CytB* using primer HCO2198 (5′-TAAACTTCAGGGTGACCAAAAAATCA-3′) and LCO1490 (5′-GGTCAACAAATCATA AACATATTGG-3′), and 12S using primer MiFish-U-F (5′-GTCG GTAAAACTCGTGCCAG C-3′) and MiFish-U-R (5′-CATAGTGGGGTATCTAATC CCAGTTTG-3′). PCR amplification was performed using a Takara PCR Thermal Cycler MP (TP3000). The PCR-amplified products were examined by 1% agarose gel electrophoresis, and a bright band under ultraviolet light indicated the target sequence. The amplified products were sent to Shanghai Personal Biotechnology Co., Ltd, and were sequenced bidirectionally using an ABI 3730XL automated sequencer. The sequences were edited and assembled into contigs using the DNAStar software package. Seqman software combined with a sequencing peak map was used to edit and correct the sequence. Published partial sequences of *COI*, *CytB*, and *12S* of all available fishes of the genus *Branchiostegus* were downloaded from Genbank (Sayers et al. 2022). Details of the downloaded data are included in Suppl. material 1.

All sequences sequenced and downloaded were aligned and concatenated into alignments using MAFFT alignment (Katoh and Standley 2013) in GENEIOUS 2022.2.2 (Kearse et al. 2012, https://www.geneious.com). Some species are missing at least one of the fragments in public repositories. Phylogenetic analysis was performed using FastTree 2.1 (Price et al. 2010) with the GTR+CAT model. For DNA-based species delimitation, a genetic distance matrix was generated based on *COI* alignment. Pairwise distances between sequences were computed on GENEIOUS.

## ﻿Taxonomic account

### ﻿ Branchiostegus sanae sp. nov.

Taxon classificationAnimaliaPerciformesMalacanthidae

314D703E-FACE-5DA7-9EBC-6D66F1377C08

https://zoobank.org/43D9907B-2D9E-482F-A533-68039CE1662D

#### Type materials.

**Figure 1.**
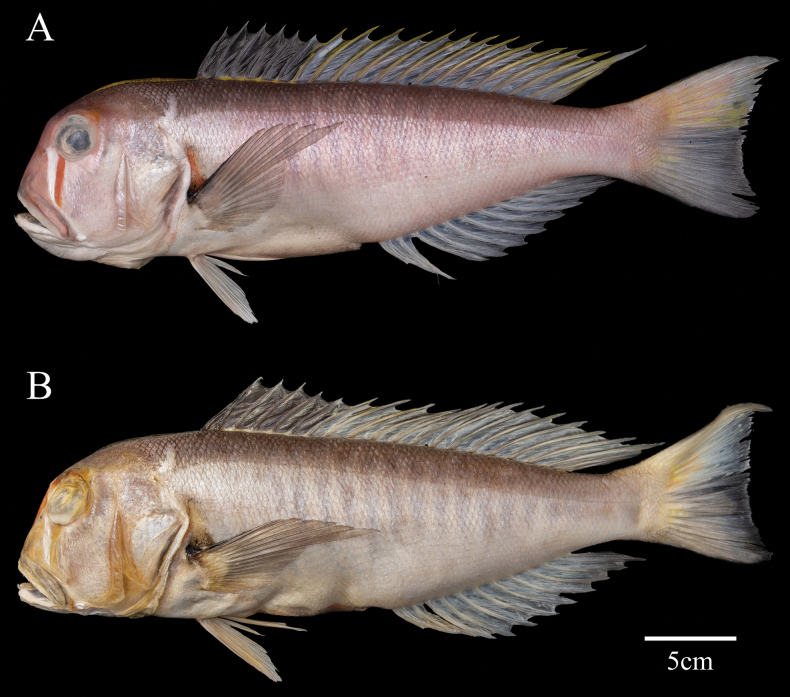
*Branchiostegussanae* sp. nov. **A** fresh specimen **B** fixed by 75% alcohol, MBM 287909, holotype, male, 350.1 mm SL, South China Sea, China.

***Holotype*.** • MBM 287909, 350.1 mm SL, male, 17.8°N, 110.5°E, northern South China Sea, China, 13 March 2023, purchased by Chi Zhang, in IOCAS (Fig. 1).

***Paratypes*.** • IOZ 233304, 313.5 mm SL, sex uncertain, 17.8°N, 110.5°E, northern South China Sea, China, 13 March 2023, purchased by Chi Zhang, in IOZ; • SCSMBC 031014, 263.9 mm SL, sex uncertain, 17.8°N, 110.5°E, northern South China Sea, China, 13 March 2023, purchased by Chi Zhang, in SCSIO; • SNHM–Hfi 13217, 295.3 mm SL, sex uncertain, 17.8°N, 110.5°E, northern South China Sea, China, 13 March 2023, purchased by Chi Zhang, in SNHM; • ZJUz 00077, 304.0 mm SL, sex uncertain, 17.8°N, 110.5°E, northern South China Sea, China, 13 March 2023, purchased by Chi Zhang, in ZJU.

#### Diagnosis.

The species can be distinguished from its congeners in having the following combination of characteristics: a unique white-red bar between the orbit and upper jaw; distal end of upper jaw vertical reaching or exceeding midpoint of orbit; yellow predorsal ridge with dark rim; dorsal fin membrane translucent greyish, with spines darker; dorsal fin spines grey and soft rays yellow; about 16 dark vertical stripes on each side; longest dorsal fin soft ray long, 20.6–22.7% in SL; no black spots on base of scales; and caudal fin nearly truncated, upper part pale orange mixed with yellow stripes, and lower part dark grey with inconspicuous yellow blotch near base.

#### Description.

Morphometric measurements and counts are summarized in Table 2. Body moderately elongate and compressed. Body depth (BD) is almost equal to or slightly shorter than head depth. Predorsal ridge (stronger in large individuals) extending vertically near center of eye. Mouth terminal, oblique; distal end of upper jaw vertical reaching or exceeding midpoint of orbit. Head moderately enlarged; anterior profile straight. Eyes very close dorsolaterally to forehead contour. Orbit diameter large (25.3–34.9% in HL), subequal to or longer than suborbital depth. Anterior nostrils tubular, with a cutaneous tongue-shaped flap on its posterior rim and located closer to the snout than anterior margin of orbit while posterior nostril oval-like without fleshy flap and located about mid-point between snout and anterior margin of the orbit. Both jaws with 3 or 4 rows of irregular canine-like teeth, with 17–20 of these canines enlarged and primarily distributed in central front and near sides. An irregular villiform teeth band on upper jaw. No teeth on palatine, vomer, or tongue. Posterior margin of preopercle serrated; a few serrations extend to ventral margin, rest of ventral margin smooth.

**Table 2.** Measurements and counts of *Branchiostegussanae* sp. nov.

	Holotype	Paratypes
Total length (mm)	416	325–368
Standard length (mm)	350	264–304
Measurements (as % in SL)		
Body depth	27.5	24.8–30.2
Body width	12.7	11.5–14.6
Head length	28.3	29.9–30.9
Head depth	27.2	24.4–26.5
Snout length	8.3	8.0–8.2
Predorsal length	31.5	30.6–31.1
Upper jaw length	11.4	11.4–13.2
Opercular length	10.0	8.1–8.4
Suborbital distance	8.9	7.0–9.1
Orbit diameter	8.1	7.8–10.4
Interorbital width	8.6	8.0–9.5
Dorsal-fin base length	56.3	52.0–57.3
Anal-fin base length	30.0	30.3–30.5
1^st^ dorsal-fin spine length	6.1	4.9–8.4
2^nd^ dorsal-fin spine length	9.7	8.8–11.5
Longest dorsal-fin spine length	10.8	9.9–12.4
1^st^ dorsal-fin soft ray length	12.8	13.0–13.4
Longest dorsal-fin soft ray length	20.6	20.7–22.7
1^st^ anal-fin spine length	4.0	2.3–3.5
2^nd^ anal-fin spine length	6.6	5.9–6.5
1^st^ anal-fin soft ray length	10.7	9.7–10.2
Longest anal-fin soft ray length	15.7	14.5–15.6
Pelvic-fin spine length	11.7	11.5–13.5
1^st^ pelvic-fin soft ray length	14.4	13.2–15.3
2^nd^ pelvic-fin soft ray length	15.0	13.6–15.2
3^rd^ pelvic-fin soft ray length	12.1	12.2–12.3
7^th^ pectoral-fin length	24.2	22.5–26.5
8^th^ pectoral-fin length	16.8	16.9–18.3
Caudal peduncle length	15.4	11.5–15.8
Caudal peduncle depth	10.3	10.5–11.0
Longest gill raker length	1.9	2.2
Length of snout to pectoral-fin origin	29.2	29.6–30.5
Length of snout to pelvic-fin origin	30.0	28.5–29.9
Length of snout to anal-fin origin	58.6	58.0–63.0
Largest cheek scale diameter	1.7	1.6–2.1
Predorsal ridge length	17.8	19.1–20.0
Predorsal ridge width	1.9	0.6–1.7
Counts		
Cheek scale rows	9	8–12
Pored lateral-line scales	47+1	44–49+1–3
Longitudinal scale rows	76	68–89
Scales above lateral line	7	6–7
Scales below lateral line	22	20–22
Gill rakers on 1^st^ arch	7+14	7–8+14
Dorsal-fin rays	VII, 15	VII, 15
Pectoral-fin rays	20	18–20
Pelvic-fin rays	I, 5	I, 5
Anal-fin rays	II, 12	II, 12
Caudal-fin rays	2+15+2	2+15+2

Cheeks, opercle, nape, and body scaled. Scales on cheek, opercle and near breast cycloid and ctenoid remain parts; 6 or 7 diagonal scale rows on cheek; scales enlarged at second, third, and fourth rows; diameter of largest cheek scales 4.7–5.0 in orbit diameter (OD). Dorsal-fin origin above pectoral-fin base; first spine shortest, length 1.0–1.9 in OD; length of 2^nd^ to 7^th^ spines almost equal, longer than 1^st^. First dorsal soft ray shortest, length 1.2–1.7 in OD, 13^th^ dorsal-fin soft ray longest 0.4–0.5 in OD, 15^th^ dorsal-fin soft ray shortest; lengths of soft rays length gradually increase from the 1^st^ to 13^th^, rapid decreases in 13^th^ to 15^th^ soft rays; dorsal-fin soft rays longer than all spines and peduncle depth except 15^th^ soft ray, only 13^th^ soft ray reaching caudal-fin base. Pectoral fin lanceolate, its base located just posterior to preopercle margin, reaching to anal-fin origin; 1^st^ to 7^th^ soft rays lengthening; 7^th^ soft ray longest, 1.1–1.4 in head length (HL); 8^th^ soft ray discontinuously shortening, 1.6–1.8 in HL; under 8^th^ soft ray, pectoral-fin soft rays evenly shortening. Pelvic-fin triangular, short, extending to midpoint of its origin to anus; pelvic-fin spine 0.7–0.9 in OD; 2^nd^ soft ray longest, 0.6–0.7 in OD. Caudal peduncle depth 2.2–2.9 in BD, shorter than its length. Caudal fin almost truncate but slightly emarginate.

When fresh, head and body are plum-colored, with ventral side lighter and dorsal aspect darker. Distinctive red vertical stripe present beneath eye, closely followed by a white band anteriorly. Snout Indian red; cheek region lighter colored. Upper part of operculum rosy brown. Plum vertical stripes taper from dorsal to ventral sides. Base of dorsal-fin spines bear gold spots, with spines and interstitial membranes dark grey. Soft rays of dorsal fin gold, yet their bases grey. Base of pectoral fins with slightly darker dark-red blotches covered by pectoral fins, and fin rays grey. Base of caudal fin paler orange, with upper 2/5 tinged with yellow and lower 3/5 dark grey. Pelvic and anal fins milky white, but distal ends of 12^th^, 13^th^, and 14^th^ anal-fin soft rays grey-black.

#### Distribution and habitat.

**Figure 2.**
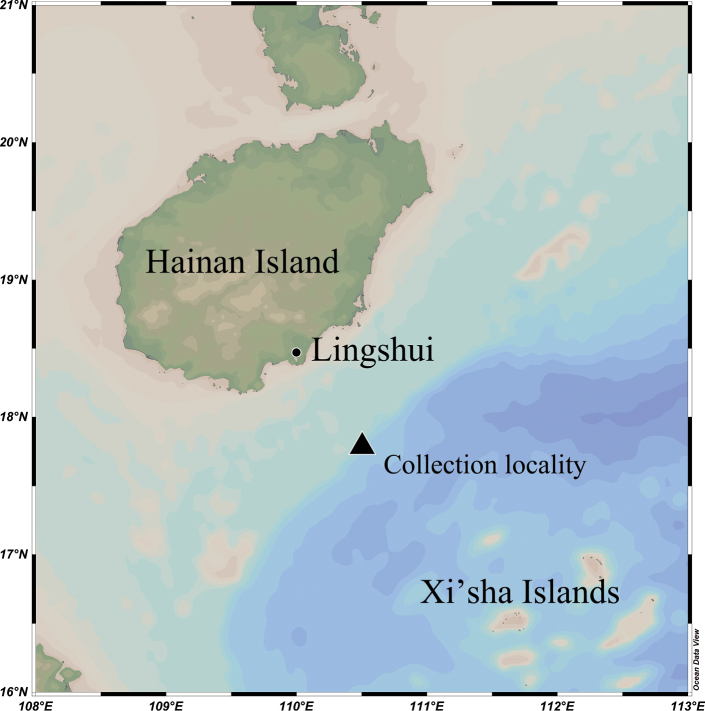
The collection location of *Branchiostegussanae* sp. nov.

The fishing area was at approximately the coordinates 17.8°N, 110.5°E in the South China Sea, on the northern slope between Lingshui, Hainan Island and Xisha Islands, at a depth of about 150–300 m (Fig. 2).

#### Etymology.

The name *sanae* refers to the heroine’s name, San in Hayao Miyazaki’s film *Princess Mononoke*, who has similar red under-eye stripes to this species and symbolizes the ideas and appeals of harmonious coexistence between man and nature that we want to share (Miyazaki 1997).

#### Common name.

Both the Chinese and English common names of this species are derived from the title of the film and align with the common name used by Chinese fishermen, “鬼马头鱼” (Ghost horsehead fish), due to the unusual cheek patterns of this species.

##### ﻿Phylogenetic analysis

**Figure 3.**
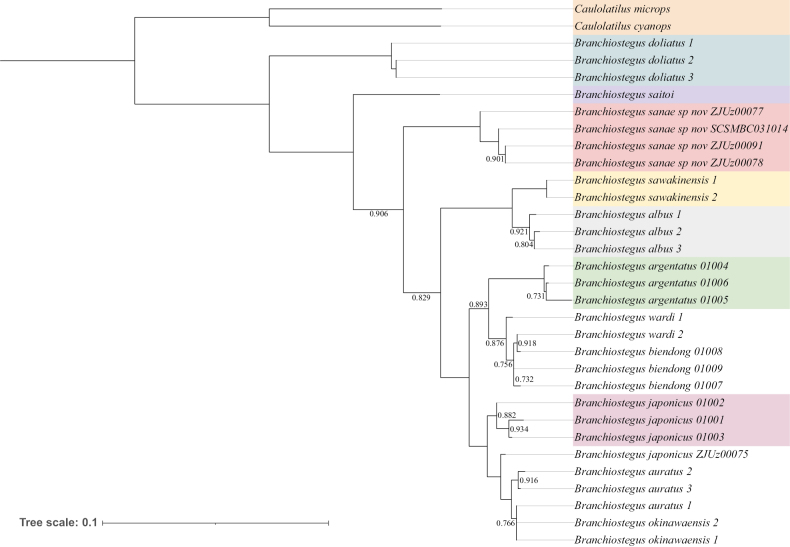
The approximately-maximum-likelihood phylogenetic tree of 10 species of the genus *Branchiostegus* and two outgroup species. The tree was constructed using concatenated sequences of *Cytb*, *COI*, and *12S* genes, totaling 1034 nucleotides. Only bootstrap values below 0.95 are displayed on the tree.

Here we present the most comprehensive molecular phylogeny of *Branchiostegus* to date, which includes 10 of the 18 known species and the new species, *B.sanae* sp. nov. (Fig. 3) The approximately-maximum-likelihood phylogenetic tree has bootstraps value exceeding 0.731 at every node in the backbone of the tree. The tree indicates that all *B.sanae* specimens we collected in this study form a monophyletic clade sister to all species but *B.saitoi* and *B.doliatus*. The southwestern Indian Ocean species *B.doliatus* appears to be a clade sister to all other species of *Branchiostegus* included in our analysis. The rest of the analyzed species occur in the western Pacific Ocean, which is the center of distribution of the genus. COI-based molecular species delimitation methods also indicate that *B.sanae* is a genetically distinct species (see Suppl. material 2).

## ﻿Discussion

To date, including this study, 19 species of *Branchiostegus* have been described. Using as reference past studies (Dooley and Rau 1982; Dooley and Kailola 1988; Hiramatsu and Yoshino 2012; Dooley and Iwatsuki 2012; Hiramatsu et al. 2019; Wu and Zhong 2021; Muhammad and El-Mahdy 2022), we have compiled a key to species of the genus as follows:

### ﻿Key to species of the genus *Branchiostegus* (* represents a nomen dubium)

**Table:** 

1	Pored lateral-line scales 67–72+2	***B.serratus*** (eastern Australia)
–	Pored lateral-line scales not exceeding 65	**2**
2	Dorsal-fin spines VI	**3**
–	Dorsal-fin spines VII	**4**
3	Anal fin spine I (rarely II), anal-fin rays 13, body with 19–20 dark vertical stripes, dark area from the angle of gill opening to pectoral-fin base	***B.semifasciatus*** (West Africa)
–	Anal-fin spines II, anal-fin rays 12, body with 16–18 dark vertical stripes, no dark area from the angle of gill opening to pectoral-fin base	***B.doliatus*** (South Africa; Mauritius and Reunion Islands)
4	Cheek scales rows 5 or 6	**5**
–	Cheek scales rows 7 or more	**6**
5	Yellow patch below orbit, dorsal-fin margin black, no black spots between dorsal-fin spine I–III	***B.biendong*** (South China Sea and East China Sea)
–	No yellow patch below orbit, dorsal-fin without margin black, a black spot between dorsal-fin spine I–III	***B.okinawaensis*** (Ryukyu Islands)
6	Dark vertical stripes on body	**7**
–	Dark vertical stripes on body faint or not apparent	**8**
7	Scales below lateral line 17, pectoral-fin without black patch, no pattern below orbit, yellow rim on dorsal-fin ray membrane, lower part pearly white	***B.gloerfelti*** (central Sumatra)
–	Scales below lateral line 20–24, black patch at pectoral-fin base, bright red stripe below anterior part of eye extending to edge of upper jaw with an adjacent white stripe, dorsal-fin rays bright yellow with grey membrane	***B.sanae* sp. nov.** (South China Sea)
8	Dorsal-fin rays 14, pored lateral-line scales 60	B.ilocanus* (Philippines)
–	Dorsal-fin rays 15, pored lateral-line scales not exceeding 55	**9**
9	Black spots on scales base near axil, dorsal-fin membrane base black	***B.sawakinensis*** (eastern Africa, Red Sea, Philippines, and northern Australia)
–	No black spot in axil, base of dorsal-fin membrane not black or dark	**10**
10	Distinct markings below orbit	**11**
–	No markings on cheek or snout	**15**
11	Three silver stripes below eye extending to the snout, upper jawbone, and lower edge of opercle	B.vittatus* (Philippines)
–	One or two silver or pearly stripes below orbit	**12**
12	Two silver or pearly stripes below orbit	**13**
–	One silver or pearly stripe below orbit	**14**
13	Jaws extend to the vertical with the midpoint of orbit, scales below lateral line 15–20, gill rakers on first arch 19–22	***B.argentatus*** (East Asia, Japan to South China Sea)
–	Jaws not reaching the vertical with midpoint of orbit, scales below lateral line 23, gill rakers on first arch 23	***B.australiensis*** (Sumatra and Australia)
14	Stripe near anterior of orbit, extending to upper jaw	***B.auratus*** (East Asia, Japan to South China Sea)
–	Stripe next to posterior of orbit, triangular, interrupted on cheek	***B.japonicus*** (East Asia, Japan to South China Sea)
15	Predorsal length 35% in SL, golden yellow patch above base of pectoral-fin, with a white patch in it	***B.saitoi*** (Philippines)
–	Predorsal length 30–32% in SL (rarely 33–34%)	**16**
16	Caudal fin uniformly colored, without distinct yellow stripes or spots	***B.albus*** (East Asia, Japan to South China Sea)
–	Caudal fin with distinct yellow stripes or yellow patches	**17**
17	Lower lobe of caudal fin with a large triangular black spot	***B.wardi*** (eastern Australia)
–	Lower lobe of tail fin without black spots	**18**
18	Cheek scale rows 7, interorbital width 28%–29% in HL	***B.hedlandensis*** (western Australia)
–	Cheek scale rows 8–11, interorbital width 21% in HL	***B.paxtoni*** (western Australia)

**Figure 4.**
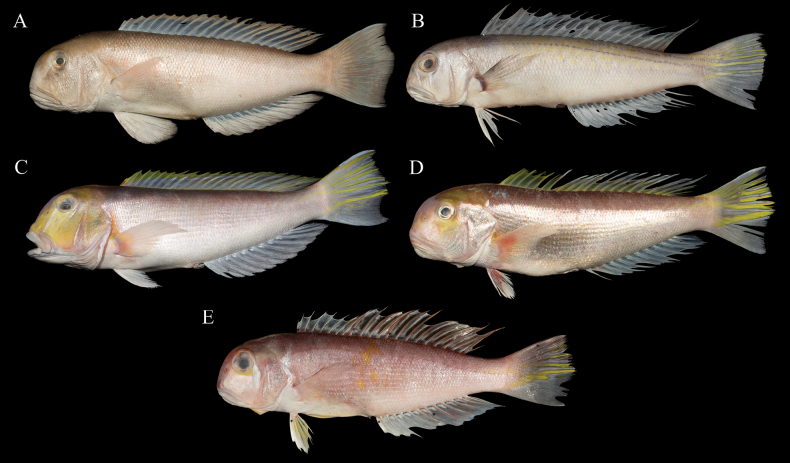
Species of the genus *Branchiostegus* in Chinese water except *B.sanae* sp. nov. **A***B.albus*, 356.3 mm SL, collected from the northern South China Sea, China **B***B.argentatus*, 263.7 mm SL, collected from the northern South China Sea, China **C***B.auratus*, 393.5 mm SL, collected from the northern South China Sea, China **D***B.biendong*, 378.0 mm SL, collected from the East China Sea, China **E***B.japonicus*, 249.7 mm, collected from Zhoushan in Zhejiang Province, China.

The genus *Branchiostegus* was established by Rafinesque (1815) and systematically reviewed by Dooley (1978). Among *Branchiostegus* species, *B.ilocanus* and *B.vittatus* are considered nomina dubia due to the possible destruction of the type specimens during World War II and the lack of subsequent records (Dooley 1978; Dooley and Iwatsuki 2012). However, recent studies have still discussed these species (Hiramatsu and Yoshino 2012; Hiramatsu et al. 2019). We retained these two species in Table 1 and the key, although with proper annotations. For many years, four species of *Branchiostegus* have been known in China: *B.albus*, *B.argentatus*, *B.auratus*, and *B.japonicus* (IOZ et al. 1962; Zhu 1985; Chen et al. 2002; Wu and Zhong 2021). Lin et al. (2016) recorded “*Branchiostegusalbus*” in the South China Sea with a photograph that can be certainly recognized as *B.sanae* sp. nov. However, the description and corresponding sequence (GenBank: KP266796) in this record match those of *B.albus* perfectly. Therefore, we do not consider that *B.albus* (Lin et al., 2016) is a senior synonym of *B.sanae* sp. nov. Actually, Lin et al. (2016) had a number of specimens and never indicated which specimen(s) the photo, description, and sequence came from. Hiramatsu et al. (2019) reported *B.biendong* as a new species occurring in the South China Sea. Including *B.sanae* sp. nov., six known species have now been recorded in China (Fig. 4). In addition, three hybrids have been reported in East Asia: *B.japonicus* × *B.auratus*, *B.japonicus* × *B.albus*, and *B.japonicus* × *B.argentatus* (Yamada et al. 2007; Yamashita et al. 2013). Beyond the type specimens, more than 20 specimens of *B.sanae* sp. nov. were collected (for other studies), all of which exhibited consistent morphological traits. Combined with phylogenetic evidence, we can exclude the possibility that *B.sanae* sp. nov. is a hybrid and differentiate it from the other species or hybrids.

Another interesting finding is that the two sequences of southwestern Pacific *B.wardi* did not form a monophyletic clade, Instead, it intercalates within *B.biendong*, which makes the latter paraphyletic. We examined the sequences and found that the two *B.wardi* CytB sequences are obtained from the *B.wardi* caught in the South China Sea, where the species has not been previously reported (Ryu et al. 2009). Therefore, further investigation of the relationship between *B.wardi* and *B.biendong* is still needed. The same situation also occurs in the clade containing *B.auratus* and *B.okinawaensis*. The *B.auratus* sample we sequenced (ZJUz00075) appear to be sister to the rest of *B.auratus* and all *B.okinawaensis*. So, this clade also deserves more attention.

In the last decade, only two new species of this genus have been described, but both of them come from the South China Sea. Reviewing the distribution of all *Branchiostegus* species, we find that all except *B.semifasciatus* are distributed in the Indo-West Pacific region. This region is also considered the center of global marine biodiversity (Xu 2021). Deepwater tilefishes are important economic fish for food (Bo et al. 2005), including *B.biendong* and *B.sanae* sp. nov. Recently, they have been sold in quantities in both online and offline seafood markets in China. The late discovery and description of *B.sanae* sp. nov. suggest our lack of knowledge about some “common species”. The diversity of the genus *Branchiostegus* may require further investigation.

## Supplementary Material

XML Treatment for Branchiostegus sanae

## ﻿Additional information

### Author ORCIDs

Haochen Huang https//orcid.org/0009-0000-6603-3874

Jingxuan Chen https//orcid.org/0009-0001-7269-4852

Zhixin Ke https//orcid.org/0000-0002-5959-737X

Chi Zhang https://orcid.org/0000-0003-3211-1410

### Data availability

All of the data that support the findings of this study are available in the main text or Supplementary Information.

## Supplementary materials

Supplementary material 1 Sequences used for phylogenetic analysis Data type xlsx Explanation note All sequences we used for phylogenetic analysis in this study. https://binary.pensoft.net/file/1247875This dataset is made available under the Open Database License (http://opendatacommons.org/licenses/odbl/1.0/). The Open Database License (ODbL) is a license agreement intended to allow users to freely share, modify, and use this Dataset while maintaining this same freedom for others, provided that the original source and author(s) are credited.Author: Jingxuan Chen

Supplementary material 2 COI based genetic distance matrix Data type csv Explanation note A COI based genetic distance matrix of eight species in genus *Branchiostegus* including *B.sanae* sp. nov. https://binary.pensoft.net/file/1247876This dataset is made available under the Open Database License (http://opendatacommons.org/licenses/odbl/1.0/). The Open Database License (ODbL) is a license agreement intended to allow users to freely share, modify, and use this Dataset while maintaining this same freedom for others, provided that the original source and author(s) are credited.Author: Jingxuan Chen
